# The Hygienic Superiority of Gas Lighting

**Published:** 1907-12-28

**Authors:** 


### THE HYGIENIC SUPERIORITY OF GAS LIGHTING.

On June 19 last Professor Vivian B. Lewes, F.I.C., F.C.S., delivered an interesting and instructive lecture before the Royal Dublin Society on "The Relative Hygienic Advantages of Gas and Electric Lighting." The subject is one which demands the attention of every householder and, incidentally, of every medical man, and Professor Lewes' conclusions are so carefully drawn, and apparently based on such searching experiments, that no apology is needed for giving them here in full. Taking ordinary dwelling-rooms lit by gas and by filament lamps respectively, Professor Lewes finds that when the rooms are occupied by two people the purity of the air in each is equal. If, however, the number of persons in the room be increased, the air of the second chamber rapidly deteriorates, much more quickly than that of the room lit by gas-burners. To many this announcement will come as a surprise. Not unnaturally, it has been assumed that electric light is inert so far as contamination of the atmosphere is concerned. It withdraws no oxygen from, and it adds no impurity to, the air of the room which is lit by it. It seems reasonable, therefore, to assume that hygienically it is superior to its competitor, gas, which vitiates the air of the dwelling-room by adding to and withdrawing from it carbon dioxide and oxygen respectively. Professor Lewes, however, lays stress on the fact that "it is the organic matter given off from the lungs and tissues during respiration that it is essential to remove from the air," and that the great factors which enable us to get rid of this organic impurity by means of ventilation "are the air currents set up by alterations in temperature and inter-diffusion between volumes of air at different temperatures."

He shows that "the combustion of 1 cubic foot of coal gas will use up 6 cubic feet of air; giving approximately half a cubic foot carbon dioxide and nearly one cubic foot and a half of water vapour. Using an incandescent mantle on an atmospheric burner, about 4 cubic feet of gas per hour are consumed, and this gives two of carbon dioxide, which would very soon suffice to raise the proportion of carbon dioxide above the sanitary limit of six parts in 10,000. But though everything be done to render the room as air-tight as possible, it will be found that the proportion of carbon dioxide is enormously less than it should be by theory, this being due to the fact that alterations in the temperature of the air of the room are set up by currents and actions which tend to bring about a change of the atmosphere."

Experimenting with the two illuminants, he obtained the following interesting results :

With two Welsbach "C" burners on pendant, each consuming 4 cubic feet of gas per hour, and of 140 candle-power, the temperature of the room at the "breathing-level" was 63.0° F. and the proportion of $CO_2$ 0.05 per 10,000. With three 16-candle-power incandescent electric lights the temperature of the same room at the same level was 61.7°F., and the amount of $CO_2$ 0.06 per 10,000.

"If a number of people are in a room, the organic exhalations as well as the carbon dioxide and water vapour evolved during respiration rise, and, reaching the level of the gas-burners, are rapidly swept up to the ceiling by the rush of hot gas from the burner—the flame and heat destroying and charring a large proportion of the germs. The hot air reaches the ceiling and diffuses through the plaster and walls in the upper part of the room, and in doing so the charred organic matter is left behind, filtered off on the surface of the plaster, and rapidly causes that discoloration of the ceiling which is invariably found in a town atmosphere above the gas-burner, and which is often wanting with country air. That this is the case is amply verified by the fact that if beams are present at the back of the plaster, diffusion is prevented at these points, and their position is plainly mapped out on the discoloured surface.

"When the room with its occupants was lighted by electric light, there was no rapid uprush in this way of the products to the ceiling; and the organic impurities and carbon dioxide leaving at body temperature remained diffused throughout the whole of the atmosphere of the room, causing a far more rapid fouling of the air and injury to health. If such a room were entirely left for its ventilation to diffusion through the walls, it would soon acquire that sour smell which is noticeable in many rooms of the poor, in which, in order to keep in the warmth derived from their own bodies, all ventilaton is cut off. This smell is due to the decomposition of organic matter filtered off during diffusion by the wall surface, and undergoing putrefactive decay, giving the offensive odour, the only way to get rid of which is to strip the paper from the walls and lime-wash them, as well as the ceiling. Then, and then only, does the smell pass away."

The superiority of gas light over electric light, from a hygienic point of view, is, however, conditioned by the position of the gas-burners. If the gas is burned at a low level, as a table light for instance, much of the "ceiling effect" appears to be lost.

In view of the fact that electric light is admittedly inferior to gas light for reading purposes, in some cases severely trying to the eyes, and that as a general rule it is a much more expensive illuminant, Professor Lewes' conclusions will certainly tend to benefit the popularity of the older competitor, which has been sensibly declining since the electric pendant came into use.

